# Association between Nephrolithiasis, Hypertension and Obesity in Polycystic Kidney Disease

**DOI:** 10.3889/oamjms.2016.010

**Published:** 2015-12-24

**Authors:** Valbona Bajrami, Alma Idrizi, Enver Roshi, Myftar Barbullushi

**Affiliations:** 1 *Diagnostic Center Ikeda, Tirana, Albania*; 2 *Service of Nephrology, UHC Mother Teresa, Tirana, Albania*; 3 *Department of Public Health, UHC Mother Teresa, Tirana, Albania*

**Keywords:** nephrolithiasis, hypertension, polycystic kidney disease, body mass index, anatomic and metabolic factors

## Abstract

**AIM:**

We aim to define the correlations between nephrolithiasis, hypertension, age and obesity in patients with autosomal dominant polycystic kidney disease (ADPKD) in Albania.

**MATERIAL AND METHODS:**

We included 100 patients with autosomal dominant polycystic kidney from 2011 to 2014. The patients underwent X-ray and renal ultrasonography. We performed the metabolic evaluation of blood and urine.

**RESULTS:**

The patients with renal stones had a higher level of mean systolic and diastolic blood pressure compared with patients without stones (155 ± 12 mmHg vs. 145 ± 8 mmHg, and 105 ± 0.9 mmHg vs. 92 ± 1.28 mmHg, respectively). Patients with renal stones were older (47 ± 15 vs. 38 ± 5 years), had a higher prevalence of obesity [body mass index (BMI): 28 ± 2.4 vs. 25.7 ± 0.6], had higher levels of total cholesterol level (220 ± 5 mg/dl vs. 203 ± 4 mg/dl) as well as triglyceride levels (160 ± 9 mg/dl vs. 126 ± 4 mg/dl), compared with no renal stone individuals.

**CONCLUSION:**

ADPKD patients with renal stones in our study had a higher mean level of systolic and diastolic blood pressure, BMI and cholesterol and triglycerides levels compared with individuals without renal stones.

## Introduction

Nephrolithiasis is an important manifestation of autosomal dominant polycystic kidney disease (ADPKD), which ranges from 8% to 36% in different studies [1-10], about twice higher than in general population.

Most ADPKD patients affected by nephrolithiasis are above 40 years old. The disease frequency begins to rise between 20-40 years, making very uncommon finding nephrolithiasis under the age of 20 years [11, 12]; even so there are studies that have found cases of nephrolithiasis in ADPKD under the age of 20 years [9, 13].

Recent reports suggest that the etiology of stone disease in ADPKD is multifactorial in nature, including both anatomic and metabolic factors, and a combination of these factors [6, 7, 9, 11, 12, 14]. Hypertension and obesity are also social diseases with important epidemiological similarities to nephroloithiasis. Various studies have demonstrated high calciuria in hypertensives with a linear relationship between 24-h calciuria and arterial blood pressure.

Also, body mass index (BMI) and body weight are independently associated with an increase in stone risk even though, due to a number of bias (limited weight categories, low number of obese persons in the study populations, no control group, no recording of food intake) the studies published failed to be conclusive.

We aim to define the correlations between nephrolithiasis, hypertension, age and obesity in patients with ADPKD in Albania.

## Material and Methods

We included 100 patients with autosomal dominant polycystic kidney from 2011 to 2014 in a prospective controlled study. The ethic committee of the Faculty of Medicine had approved the study. The patients underwent X-ray and renal ultrasonography. We performed the metabolic evaluation of blood and urine. The diagnosis for ADPKD is done based on criteria established by Ravine et al. in 1994 [15]: the presence of three or more (unilateral or bilateral) renal cysts for individuals aged between 15 to 39 years, two or more cysts in each kidney for individuals aged 40 to 59 years, and four or more cysts in each kidney for individuals over 60 years old.

All patients underwent renal ultrasound to determine cyst number and predominant cyst size. Patients with nephrolithiasis were defined as those with stones within the collecting system. To diagnose renal stones we used all imaging methods, renal ultrasound (identification of an echogenic focus with posterior acoustic shadowing within the kidney), plain abdominal kidney–ureter–bladder film (KUB) for radiopaque stones, intravenous pyelography (more rarely), which can provide both anatomical and functional information on stones and the urinary tract and non-contrast helical computed tomography (CT) scan in cases when nephrolithiasis was not observed by KUB or renal ultrasound.

Subjects were considered normotensive if they had not taken medication for hypertension, the mean of three systolic reading was less than 140 mmHg, and the mean of three diastolic reading was less than 90 mmHg at baseline [16, 17].

All variables are presented as mean ± SD. Differences were considered significant at the p < 0.05 levels.

Pearson’s correlation was used in order find the associations between diastolic blood pressure and kidney size.

## Results

Nephrolithiasis was present in 58 of our patients with ADPKD (58%). Thirty-nine patients with kidney stones were women (Table 1). The stones were composed primarily of urate (47%) and calcium oxalate (39%), and other compounds 14%. Sixty-six per cent of patients with nephrolithiasis (38 patients) had hypertension and 40% of them had increased BMI. The patients with renal stones had a higher level of mean systolic and diastolic blood pressure compared with patients without stones (155 ± 12 mmHg vs. 145 ± 8 mmHg, and 105 ± 0.9 mmHg vs. 92 ± 1.28 mmHg, respectively). Patients with renal stones were older (47 ± 15 vs. 38 ± 5 years), had a higher prevalence of obesity (mean BMI: 28 ± 2.4 vs. 25.7 ± 0.6), had higher levels of total cholesterol level (220 ± 5 mg/dl vs. 203 ± 4 mg/dl) as well as triglyceride levels (160 ± 9 mg/dl vs. 126 ± 4 mg/dl), compared with no renal stone individuals. Ten patients were with diabetes mellitus; from there 6 patients were with stones from uric acid and all of them with increased BMI.

**Table 1:** Demographic data of patients

	Patients with nephrolithiasis (58 patients)	Patients without nephrolithiasis (42 patients)	*P* value
Age	47 ± 15 years	38 ± 5 years	<0.05

Sex		
Females/Males	39/19	19/23	NS

Area of origin		
Rural area/Citizen area	40/18	28/14	<0.001

Renal function			
GFR≥60 ml/min/GFR<60 ml/min	42/16	30/12	NS

BMI (kg/m^2^)	28 ± 2.4	25.7 ± 0.6	<0.05

Smoking (Yes/No)	18/40	19/33	NS

Mean blood pressure values (mmHg)			
Mean systolic pressure	155 ± 12	145 ± 8	<0.05
Mean diastolic pressure	105 ± 0.9	92 ± 1.28	

GFR- glomerular filtration rate, BMI- body mass index, NS- not significant.

The kidney size (longitudinal diameter) was significantly greater in the hypertensive patients compared with those normotensive (16.31 ± 1.7 cm vs. 12.4 ± 1 cm, p < 0.039) (Table 2). Systolic and diastolic blood pressure correlated with kidney size (p < 0.05; r = 0.55; r = 0.63). Also, mean renal volume was significantly greater in the hypertensive patients versus the normotensive patients (580 ± 41 cm^3^ vs. 360 ± 42 cm^3^, p < 0.005) (Table 3).

**Table 2:** The correlation of hypertension with kidney size and renal volume

	Hypertensive patients	Normotensive patients	Pearson’s correlation	*P* value
Kidney size	16.31 ± 1.7 cm	12.4 ±1 cm	r=0.60	<0.039

Mean renal volume	580 ± 41 cm3	360 ± 42 cm3	r=0.75	<0.005

**Table 3:** The correlation of blood pressure with kidney size

Blood pressure	Mean ± SD	Kidney size	Pearson’s correlation	P value
Systolic blood pressure	155 ± 12	16.8 ± 1.2 cm	R = 0.55	0.04

Diastolic blood pressure	105 ± 0.9	11.5 ± 0.9 cm	R = 0.63	0.03

## Discussion

Our study confirmed that incidence of stone disease is greater in hypertensives than in normotensives also in ADPKD patients. By the same token, the incidence of hypertension is greater in stone formers than in non stone formers, but it is not clear whether nephrolithiasis is a risk factor for hypertension or vice versa. It has been suggested a relationship between structural deformation and hypertension in the ADPKD patients [18]. In the present study, hypertension was associated with greater renal structural abnormalities. Specifically, the hypertensive ADPKD patients have greater renal volumes and cystic involvement than well-matched normotensive ADPKD patients. This supports the hypothesis that cyst decompression has been associated with a decrease in blood pressure and an improvement of renal function [19, 20].

Metabolic factors are equally important in the stone forming process, as are anatomical anomalies in ADPKD. Gambaro et al. observed that metabolic disturbances were more frequent in stone-forming patients with renal anatomical anomalies [7].

Uric acid and calcium stones are the most frequent types of stones in ADPKD patients even though hyperuricosuria and hypercalciuria do not occur consistently in ADPKD patients [6, 10]. Umbreit et al. in his study also found that 55% of treated stones in ADPKD were primarily uric acid calculi [21]. Meanwhile, Daudon found that uric acid stones are the predominant type in cystic renal abnormalities [22]. The reason for this predominance of uric stones in this kind of patients is still a debate because of the low prevalence of hyperuricosuria (18-28%) in ADPKD with normal renal function [4, 13], and more over the lack of statistically difference in uricosuria between ADPKD patients with or without lithiasis [9]. The metabolic abnormalities in these patients include hypocitraturia, hyperuricosuria, hyperuricemia and presence of diabetic patients among them with stones from uric acid confirm the relationship between diabetes, hyperuricemia, metabolic syndrome, increased BMI and nephrolithiasis.

On the other hand, in the high frequency of nephrolithiasis in ADPKD patients contribute some non intrinsic factors such socioeconomic status of patients, geographic zones and dietary habits (most of them were from rural areas, consuming less water, more vegetable and animal proteins) [13].

In the final analysis, stone disease, arterial hypertension and excess weight/obesity prove to be closely interconnected and it is possible to intervene with targeted diets aimed at reducing the risk of illness and death from these diseases.

In conclusion, future studies in Albania should include larger population-representative samples. The association between nephrolithiasis and hypertension in patients with ADPKD is important in our patients. Except anatomic and metabolic factors, there are other contributor factors to this association like dietary habits. Both hypertension and stones might be addressed through lifestyle modification to prevent weight gain.
